# A flexible and stretchable photonic crystal sensor for biosensing and tactile sensing

**DOI:** 10.1016/j.heliyon.2022.e11697

**Published:** 2022-11-17

**Authors:** Wang Peng, Bing Huang, Xuanxuan Huang, Han Song, Qingxi Liao

**Affiliations:** a College of Engineering, Huazhong Agricultural University, Wuhan, 430070, China; b Shenzhen Branch, Guangdong Laboratory for Lingnan Modern Agriculture, Genome Analysis Laboratory of the Ministry of Agriculture, Agricultural Genomics Institute at Shenzhen, Chinese Academy of Agricultural Sciences, Shenzhen, 518000, China; c Shenzhen Institute of Nutrition and Health, Huazhong Agricultural University, Wuhan, 430070, China; d Key Laboratory of Agricultural Equipment in Mid-Lower Yangtze River, Ministry of Agriculture and Rural Affairs, Wuhan, 430070, China; e Technical office, No. 78170 factory, Chengdu, 610504, China; f School of Mechanical and Electronic Engineering, Wuhan University of Technology, Wuhan 430070, China

**Keywords:** Flexible and stretchable photonic crystal, Tactile sensing, Biosensing, Nanoreplica molding

## Abstract

Biosensing and tactile sensing are considered to be essential functions for intelligent diagnostic medical robot. In this paper, biosensing and tactile sensing had been demonstrated with a single photonic crystal structure. The flexible and stretchable photonic crystal structure consists of PDMS as the flexible substrate and TiO_2_ as the guided layer, and the nanograting structure was realized by nanoreplica molding. For biosensing experiment, a sensitivity of 93 nm/RIU is verified with ambient environment RI variance simulation results. For tactile sensing experiment, the highest resolution for strain sensing is 0.1%, and the minimum detected scale of the grating period variation is 0.1 nm. The TiO2/PDMS structure based flexible and stretchable photonic crystal sensor demonstrates highly sensitivity and potentially cost effective for biosensing and tactile sensing, and it is promising in the area of intelligent diagnostic medical robot.

## 1 Introduction

Photonic crystals refer to periodic nanostructures composed of dielectric material layers with various refractive index materials, the combination of which can modulate the direction of light propagation [1], [2], [3], [4], [5]. A photonic crystal is generally formed by two layers of dielectric materials with different refractive indexes, in which the upper layer is characterized by a relatively high refractive index coefficient to obtain resonance coupling [6], [7], [8]. In recent years, with the excellent properties of photonic crystals in optics and electromagnetics, it has gradually developed into a popular direction of research and development [9], [10], [11]. Photonic crystal refers to an artificial periodic dielectric structure with the characteristic of photonic band gaps. The photonic band gap refers to the fact that the periodic structure prohibits the propagation of electromagnetic waves within a certain frequency range. Which is to say, the periodic dielectric structure itself has a “gap”. Photonic crystals can change the electromagnetic field according to their interaction with electromagnetic waves and the surrounding environment, thereby shifting the frequency of its resonant spectral information. Photonic crystal is a micro-nano-scale optical device, which has excellent characteristics such as small consumables, high integration, high stability and reliability, and the manufacturing process can allow large-scale production. Detection, genetic cell engineering and other fields have broad application prospects [12], [13], [14], [15], [16].

As photonic crystal sensors boast high sensitivity, high stability, easy integration, and high manufacturability, they are widely used in emerging fields such as life sciences, genetic engineering, medical analysis, and biomedicine [17], [18], [19], [20]. Conventional photonic crystal structure devices use rigid materials such as SiO_2_ and Si as the base layer, thus devices are difficult stretch, bend, or undergo elastoplastic deformation [21], [22], [23]. In tactile-oriented multi-modal sensing systems, detection devices must classify and sense different external physical quantities (e.g., pressure, strain, and temperature), conform to irregular surface substrates, and withstand large physical deformation and other functions, in addition to providing real-time and accurate perception [24], [25], [26], [27], [28]. The flexible design and manufacture of a photonic crystal structure are the key to realizing the application of flexible photonic technology in the field of robot multi-modal tactile perception.

The realization of multi-modal tactile sensing based on flexible and stretchable photonic crystals requires the ability to classify and sense different external physical parameters (e.g., pressure, strain, and temperature) simultaneously. At present, flexible and stretchable photonic crystal structures used to sense external physical quantities are mainly based on the structural mode of the optical waveguide [29], [30]. In various studies, researchers had used optical waveguide structures to test the output light intensity change as a reference quantity and measured physical quantities such as pressure and strain [31], [32]. In recent years, many new materials have been developed in the fabrication of flexible and stretchable photonic sensors [33], [34], [35], [36]. The common characteristics shared between these new materials include transparency, flexibility, and stretchability. Stretchable and flexible materials are capable for a wide range of applications that cannot be used with traditional hard materials [37], [38], [39], [40], [41], [42]. Researchers had made some staged research progress in the field of photonic crystal as biological detection or tactile perception respectively, while there are seldom work about integrated the multifunction with a single photonic crystal chip. Simultaneous realization of biosensing and tactile sensing with a single photonic crystal chip has practical application significance.

In this paper, a flexible and stretchable photonic crystal structure for biosensing and tactile sensing has been proposed, which have a huge potential in the area of intelligent diagnostic medical robot. Flexible materials with optical transparency such as PDMS are used as the stretchable base layer, and high refractive index materials such as TiO_2_ are used as the effective layer. A nano-grating structure can be sculpted on the stretchable substrate by the nanoreplica molding method, and then the high refractive index grating layer is coated by spin coating. The sensitivity of the flexible and stretchable photonic crystal is analyzed through a spectrum shift in the resonance wavelength. When parameters of the flexible photonic crystal are subjected to change such as pressure, strain, temperature, or humidity, its structure undergoes a certain physical deformation which occurs the changes of causes the grating period of the flexible photonic crystal. The changes in the grating period cause the frequency shift of the resonance peak in the photonic crystal resonance spectrogram. Therefore, the change in pressure, strain, temperature, or humidity can be determined by the frequency shift value of the resonance peak. Finally, the biosensing and tactile sensing results are analyzed to construct a high-performance flexible and stretchable photonic crystal sensor.

## 2 Physical principle and simulation of biosensing and tactile sensing

### 2.1 Physical principle and simulation of biosensing

The materials commonly used in the preparation of a traditional photonic crystal base layer include hard materials such as SiO_2_, which render traditional photonic crystal structures rigid after the design and preparation of the structures are completed. Additionally, the traditional MEMS (Micro-Electro-Mechanical System) fabrication process is costly and complicated for PC fabrication, and the grating period is fixed for a specifically designed structure. The resonance wavelength *λ* is determined by the grating height d, period Λ, duty cycle f, and polarization direction of incident light as follows [43]:

(1) $$\frac{2\pi }{\lambda }n_{c}sin\theta \pm m\left(\frac{2\pi }{\Lambda }\right)=\frac{2\pi }{\lambda }n_{eff},$$

where $n_{c}$ is the ambient environment refractive index. *θ* is the incident angle, m=0,±1,… is the diffraction order, and $n_{eff}$ is the effective refractive index of the photonic crystal. In theory, any specific wavelength can be obtained by adjusting the periodic structure of the photonic crystal.

The effective refractive index is the comprehensive result of the ambient environment refractive index $n_{eff}$, the high refractive index layer, and the low refractive index layer as follows [44]:

(2) $$n_{eff}^{2}=\frac{\int_{-\infty }^{+\infty }\int_{0}^{\Lambda }\varepsilon (x,y){\left|E(x,y)\right|}^{2}d_{x}d_{y}}{\int_{-\infty }^{+\infty }\int_{0}^{\Lambda }{\left|E(x,y)\right|}^{2}d_{x}d_{y}},$$

where E(x,y) and ε(x,y) are the electrical field value and dielectric permittivity of two dimensional distribution of PC, respectively.

**Figure 1:**
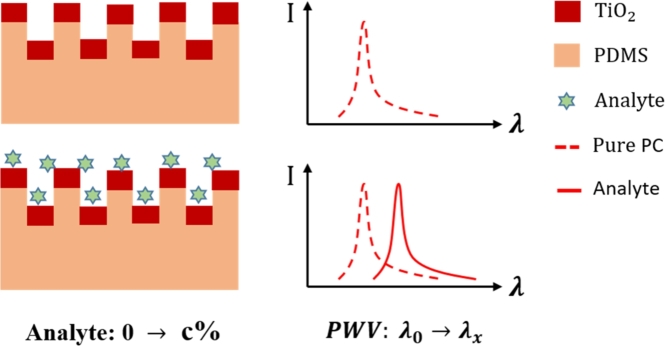
Schematic diagram of the flexible and stretchable photonic crystal for biosensing.

When the external environment is stable, the resonance wavelength generated by the traditional photonic crystal structure is a fixed value. In addition, the frequency shift of the resonance wavelength is related to the concentration of analytes, as shown in Fig. 1. When the photonic crystal structure is fixed, the relationship between the analyte concentrations (which causes changes in refractive index $\Delta n_{eff}$) and the photonic crystal resonance wavelength shift Δ*λ* has the following relationship with the grating period Λ as follows [45]:

(3) $$m\Delta \lambda =\Lambda \Delta n_{eff}$$

**Figure 2:**
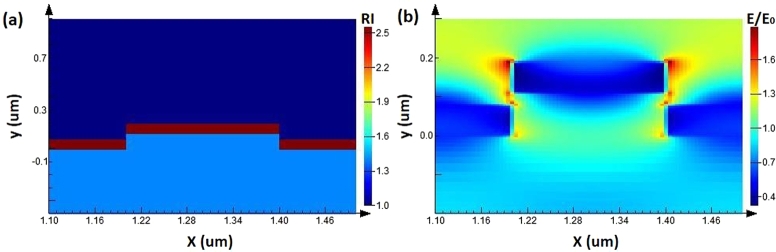
Electric field simulation of the flexible and stretchable photonic crystal: (a) Refractive index distribution of the photonic crystal; (b) Electric field distribution of the photonic crystal.

The sensing performance is determined by the structural parameters of the photonic crystal. To obtain a feasible photonic crystal structure, the material of the photonic crystal film layers, the refractive index coefficient, and the characteristic parameters of the photonic crystal (waveguide layer thickness, grating height, grating period, grating height, and duty cycle) are optimized through an electric field simulation using Finite Difference Time Domain (FDTD, Lumerical Inc.). By selecting a period of the photonic crystal structure as the simulation area and after setting the material and refractive index of each layer of the photonic crystal ($n_{TiO_{2}}=2.55,n_{PDMS}=1.40,n_{c}=1.0$), the refractive index distribution can be obtained, as shown in Fig. 2(a). The parameters are set as follows: grating period Λ = 400 nm; duty cycle = 0.5; TiO2 film thickness = 70 nm; and PDMS grating depth = 110 nm. The localized electric field distribution is shown in Fig. 2(b), and it can be observed from the figure that the enhanced electric field can fully interact with the analytes on the grating surface to achieve biosensing.

### 2.2 Physical principle and simulation of tactile sensing

By using a flexible substrate, the photonic crystal structure can be stretched and deformed when it is influenced by an external physical environment, thereby causing a relative change in the period of the photonic crystal structure. The tactile sensing performance is determined by the structural parameters of the photonic crystal. It can be concluded that when the effective refractive index coefficient $n_{eff}$ is constant, the resonance wavelength shift Δ*λ* is linearly related to the grating period variation ΔΛ [46]:

(4) $$m\Delta \lambda =\Delta \Lambda n_{eff}$$

Therefore, when the external tensile strain S causes the changes in the photonic crystal grating period [47]:

(5) $$S=\frac{\Delta \Lambda }{\Lambda }$$

According to Equation (4) and (5), the relationship between the applied external tensile strain and the resonance wavelength shift can be derived as:

(6) $$S=\Delta \lambda \frac{m}{\Lambda n_{eff}}$$

Therefore, the applied external strain S can be measured through the flexible and stretchable resonance wavelength shift Δ*λ*.

**Figure 3:**
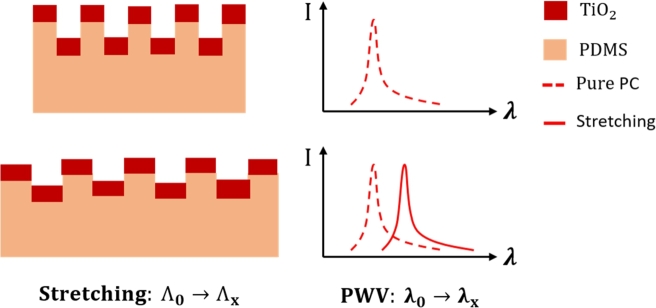
Schematic diagram of the flexible and stretchable photonic crystal for tactile sensing.

The physical deformation of a flexible and stretchable photonic crystal device can be used to detect external physical information such as pressure, strain, temperature, and humidity. The principle of flexible and stretchable photonic crystal tactile sensing is shown in Fig. 3. When the tactile sensing device is stretched and deformed by the external environment, the grating period of the flexible and stretchable photonic crystal structure changes from $\Lambda_{0}$ to $\Lambda_{x}$, and the photonic crystal resonance wavelength *λ* is shifted from $\lambda_{0}$ to $\lambda_{x}$; thus, the external stress and strain can be sensed and expressed by a shift in the resonance wavelength.

When a tactile sensing device based on a flexible and stretchable photonic crystal is used for tactile sensing, there is a situation that the stress concentration causes the device damaged or not work properly. This is because the Young's modulus of the base layer and the high refractive index functional layer are inconsistent. Therefore, it is necessary to analyze the distribution of internal strains in the structure during the fabrication of the flexible and stretchable photonic crystal device.

**Figure 4:**
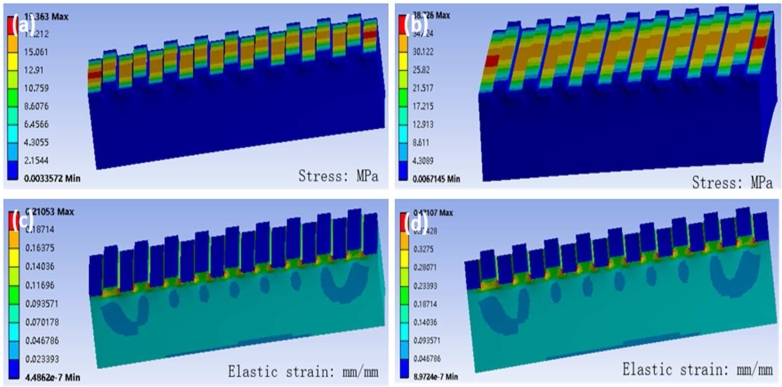
Finite element simulation results of the flexible and stretchable photonic crystal: (a) Stress distribution with 5% stretching tensile applied; (b) Stress distribution with 10% stretching tensile applied; (c) Strain distribution with 5% stretching tensile applied; (d) Strain distribution with 10% stretching tensile applied.

The basic parameters of the photonic crystal structure are: grating period = 400 nm; duty cycle = 0.5; and TiO2 layer thickness = 90 nm. The ABAQUS software (Dassault Systemes Inc.) is used for the simulation. The Young's modulus of PDMS and TiO2 is set to 1 MPa and 720 GPa, respectively, and the mass density of PDMS and TiO2 is set to 0.98 g/cm^3^ and 4.26 g/cm^3^, respectively. When stretching the flexible and stretchable photonic crystal structure by 5% along the direction perpendicular to the nano-grating, the stress distribution is as shown in Fig. 4(a). It can be seen from Fig. 4(a) that the stress is mainly concentrating on the surface of the high refractive index film, and the distribution of the stress value ranges from 0 MPa to 17 MPa. The stress distribution does not cause damage to the photonic crystal structure when the flexible and stretchable photonic crystal structure is stretched or deformed. The strain distribution obtained is shown in Fig. 4(c). It can be observed in Fig. 4(c) that the strain generated inside the flexible and stretchable photonic crystal structure is very small, and the strain is generally concentrating at the corners. When the entire flexible and stretchable photonic crystal structure is further stretched (tensile strain 10%), the stress distribution is obtained as shown in Fig. 4(b), which shows that the stress distribution is consistent with those of stretching the structure by 5% and that the corresponding stress values demonstrate a two-fold linear increasement. Similarly, the strain distribution diagram shows good consistency with that for the 5% stretch and the strain values also demonstrate a two-fold linear increase, as shown in Fig. 4(d). The finite element analysis results show that during the stretching process of the flexible and stretchable photonic crystal, its stress and strain distribution are stable, and the stress and strain value rise linearly with the increase of tensile strength. The film on the surface of the photonic crystal structure keeps the same condition during the stretching process.

## 3 Design and fabrication

**Figure 5:**
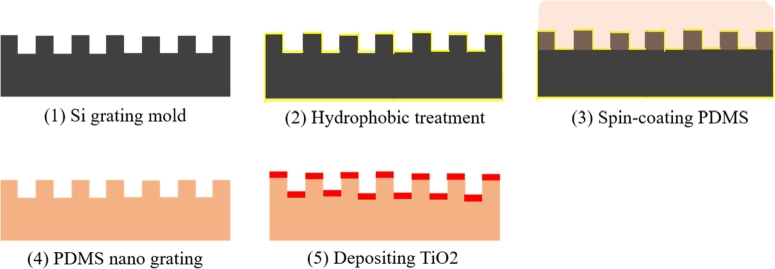
The nanoreplica molding fabrication process of a flexible and stretchable photonic crystal.

Because the flexible and stretchable photonic crystals have periodic grating structures with multilayer films, the flexible and stretchable nano-grating structure is of key importance for tactile sensing. The flexible and stretchable photonic crystal structure must be realized with periodic nanostructure features on a dielectric material and deposited on a flexible grating substrate. Instead of using e-beam etching, deep ultraviolet lithography, or embossing with a template, nanoreplica molding is used to prepare the periodic nano-grating structure. However, it is difficult to achieve nano-grating patterns on flexible materials in traditional etching processes due to the high cost and stringent process environment requirements. To realize low-cost fabrication, nanoreplica molding is used to create the flexible and stretchable photonic crystal structure. The schematic diagram of the flexible and stretchable photonic crystal fabrication process is shown in Fig. 5. The fabrication process of flexible and stretchable photonic crystal can be divided into the following 5 stages: 1) Preparation of the silicon grating template: e-beam lithography is used to create the grating pattern with a period of 400 nm, which is used as the silicon grating template. The period, grating height, and duty cycle of the flexible and stretchable photonic crystal are all determined by the grating template. 2) Surface treatment of the silicon template: the prepared silicon template is soaked in hydrophobic silane for 5 minutes, then it is taken out and cleaned with IPA (isopropanol) and dried with nitrogen, which causes the silicon wafer grating template to be hydrophobic. 3) Spin-coating PDMS: uncured PDMS and a curing agent are uniformly mixed at a ratio of 10:1 and then spin-coated onto the silicon grating template. 4) PDMS nano-grating: the PDMS and silicon grating template are placed in a vacuum oven for 30 minutes at 85°C and then the cured PDMS is peeled off from the silicon grating template. 5) Deposition high refractive index grating film layer: e-beam evaporation is applied to deposit a thin layer of TiO2 on the surface of the PDMS nano-grating substrate.

**Figure 6:**
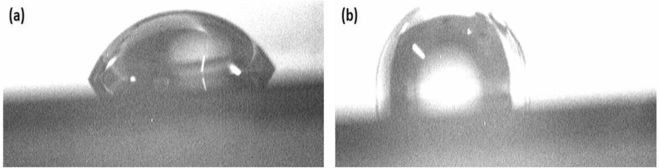
Hydrophobic treatment of the flexible stretchable photonic crystal: (a) Original Si grating template, CA 60°; (b) After hydrophobic treatment, CA 98.5°.

In the fabrication process, the peeling quality of the cured PDMS and the silicon grating template determines the performance of the prepared flexible and stretchable photonic crystal sensor. Because both the PDMS and silicon wafer surfaces are hydrophilic, it is difficult to achieve stable peeling of PDMS from the silicon wafer template. To facilitate the stable shaping and peeling of the PDMS grating, the surface of the silicon grating template is modified. The original surface of the silicon wafer showed hydrophilic properties with the test experiment showing that its contact angle (CA) was 60°, as shown in Fig. 6(a); after the surface of the silicon wafer was soaked and hydrophobicized with Repel Silane, the test experiment showed that the contact angle increased to 98.5°, as shown in Fig. 6(b). Therefore, it can be concluded that the surface of the silicon template is hydrophobic after surface modification. As the silicon template became hydrophobic, the adhesion force between the cured PDMS and the grating silicon template became smaller. Thus, it is easy to peel the cured PDMS from the silicon template. Also, as the silicon template became hydrophobic, there will be seldom PDMS polymer residue on the surface of the temple and the template can be reused for many nanoreplica molding cycles.

**Figure 7:**
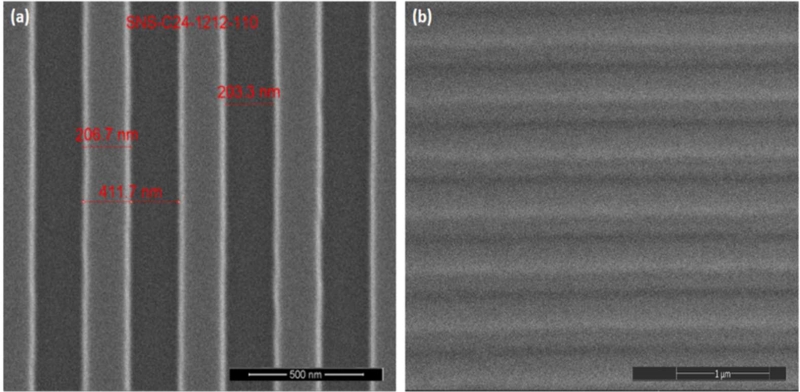
SEM images of nanoreplica molding gratings: (a) Si grating master; (b) PDMS grating.

After hydrophobizing the surface of the silicon template, the cured PDMS and the silicon grating template showed a better peeling effect. After preparing the PDMS grating substrate, the profile of the silicon grating template and the PDMS grating were compared by SEM, as shown in Figs. 7(a) and (b). It can be observed that the PDMS grating structure has the same grating period and duty cycle as those of the silicon grating template. This experiment proved that by hydrophobizing the surface of the silicon template, the peeling quality of PDMS and the silicon grating template can be improved.

## 4 Sensing experiment

A transmission spectroscopy experimental platform is used to analyze the resonance wavelength peak. Because the deformation produced by the photonic crystal structure can be detected in real time without hysteresis or electromagnetic interference, it can be used in the area of high precision measurement for tactile sensing and biosensing.

### 4.1 Tactile sensing experiment

**Figure 8:**
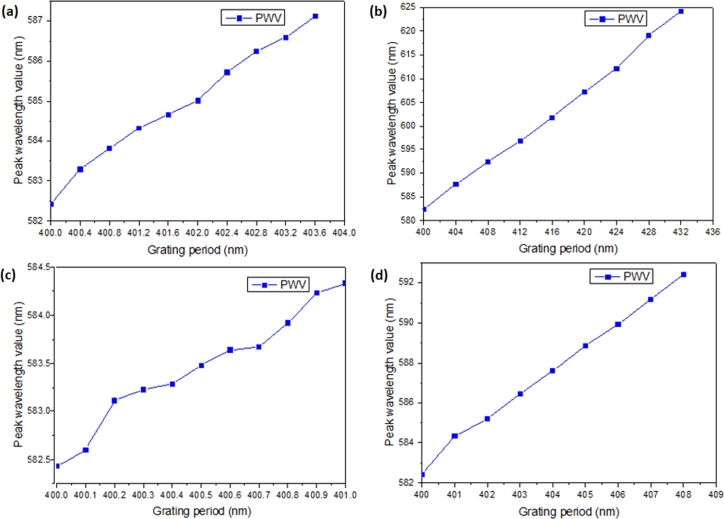
Tactile sensing of the flexible and stretchable photonic crystal: (a) 0.1% strain applied per step; (b) 1% strain applied per step; (c) Grating period increased by 0.1 nm per step; (d) Grating period increased by 1 nm per step.

The tactile sensing of the flexible and stretchable photonic crystal is divided into two aspects: 1) the sensitivity response to the grating period variation; 2) tactile sensing. When a strain of 0.1% (corresponding to a stretching distance of 0.4 nm) is applied to the flexible and stretchable photonic crystal, the peak wavelength value shift is obtained as shown in Fig. 8(a). It can be observed from Fig. 8(a) that the resonance wavelength red-shifts as the strain gradually increases, and the resonance wavelength shift value and the strain increase show good linearity. When the strain increases from 0 to 1%, the peak resonance wavelength value (PWV) of the photonic crystal red-shifts from 582.5 nm to 587 nm. Fig. 8(b) shows the relationship between the peak resonance wavelength and the strain increment when the strain on the flexible and stretchable photonic crystal increases from 0 to 8%. A good linear relationship can be observed between the resonance wavelength shift of the photonic crystal and the external strain. When the external strain increases from 0 to 8%, the resonance wavelength of the flexible and stretchable photonic crystal red shifts from 582.5 nm to 625 nm. Furthermore, although the deformation of the flexible and stretchable photonic crystal is focused on a single grating period scale, the relationship between the variation of the grating period and the peak resonance wavelength further reveals the strain sensitivity. When the grating period increases by 0.1 nm, the corresponding peak resonance wavelength shift is obtained as shown in Fig. 8(c). As the grating period of the flexible and stretchable photonic crystal is increased by 0.1 nm, the change in the grating period is linearly related to the peak resonance wavelength. Tuning the grating period of the flexible and stretchable photonic crystal from 400 nm to 401 nm results in an increase in the peak resonance wavelength from 582.5 nm to 584.3 nm. Fig. 8(d) shows the corresponding peak resonance wavelength shift when the grating period increases by 1 nm and that the change in the grating period is linearly related to the peak resonance wavelength of the photonic crystal. As the grating period increases from 400 nm to 408 nm, the peak resonance wavelength increases from 582.5 nm to 592.4 nm. From the above experiments, it can be concluded that the flexible and stretchable photonic crystal device can be used for tactile sensing. A good linear relationship is consistently demonstrated between the peak resonance wavelength shift and the applied external strain.

### 4.2 Biosensing experiment

The realization of a flexible and stretchable photonic structure can not only be applied to the field of robotic perception to achieve the sensing of multiple physical quantities (such as stress, strain, temperature, and humidity), but also used for analysis and detection in the fields of medical health and biochemistry. For a certain analyte, its refractive index is proportional to its concentration varies. When analytes are placed on the nano-grating surface of a flexible and stretchable photonic crystal, a red shift occurs in the peak resonance wavelength due to the change in the effective refractive index. Based on this principle, the flexible and stretchable photonic crystal structure can also be applied for biosensing.

**Figure 9:**
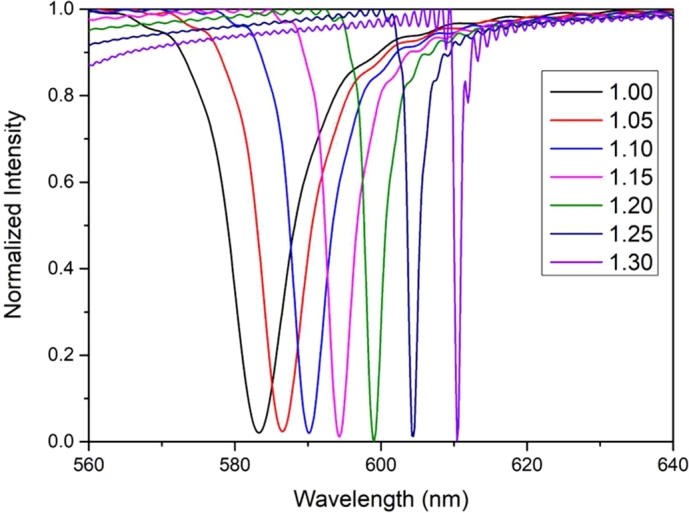
Transmission spectrum of ambient environment refractive index variation.

According to Equation (2), when a collimated and polarized light is incident into the photonic crystal structure in perpendicular direction, a specified spectral wavelength will reflect back and all the other light will pass through. In the transmission spectrum, the dip is the resonance wavelength peak. According to Equation (4), the increase in the refractive index of the analyte sample will change the effective refractive index of the photonic crystal structure, which will cause the shifting of the resonant peak in the spectrum. As the refractive index of the nano-grating surface is tuned from 1.00 to 1.30, the peak resonance wavelength in the transmission spectrum shifts from 582.5 nm to 610.4 nm, as shown in Fig. 9. Because different samples have a specific dielectric coefficient, and dielectric coefficients can modify the effective refractive index, the flexible and stretchable photonic crystal can be used to detect different analytes. Additionally, various concentrations of the analyte sample result in different effective refractive index, thus lead to a change in the resonance wavelength. Therefore, a PC-based biosensor can also be used for concentration detection. The reason about the expanding in the full width at half maximum with the increase in the index of refraction of the ambient environment, may originate from the coupling effect of guided mode resonance weakens as the refractive index difference between guided mode layer and ambient environment increases.

**Figure 10:**
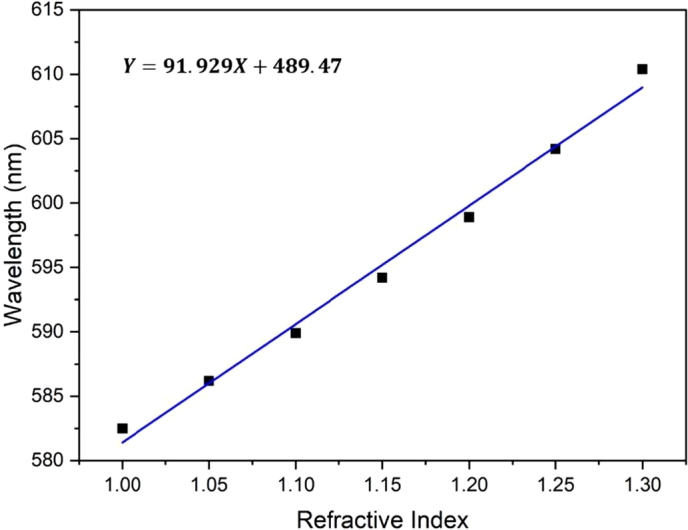
The refractive index sensitivity of the flexible and stretchable photonic-crystal-based biosensor.

As the peak resonance wavelength of a flexible and stretchable photonic crystal shifts based on the variation in ambient environment refractive index, the refractive index sensitivity of the biosensor can be calculated. When the refractive index is tuned from 1.00 to 1.30, the total shift distance of the resonance wavelength is 27.9 nm, as shown in Fig. 10. Thus, the refractive index sensitivity of the flexible and stretchable photonic-crystal-based biosensor is 93 nm/RIU (Refractive Index Unit).

## 5 Conclusion

In conclusion, a flexible and stretchable photonic crystal device is designed and fabricated with the nanoreplica molding method in this paper. In addition to applications in biochemical analysis and detection, the flexible and stretchable photonic crystal sensor can also be applied for external interaction perception (such as stress, strain, temperature, and humidity). The novelty of our work as follows: 1) Simultaneous realization of biosensing and tactile sensing with a single photonic crystal structure has been proposed, while traditional photonic crystals just have single function mode [48]; 2) flexible optical materials (PDMS) have been used to realize flexibility and stretchability of the photonic crystal, while traditional photonic crystals are made of rigid materials [49]. For tactile sensing, there is a linear relationship between the applied strain and peak resonance wavelength shift. When the flexible and stretchable photonic crystal is stretched by 0.1%, the resonance wavelength shifts 0.4 nm. For biosensing, the variation in the ambient environment refractive index is consistent with the resonance wavelength shift of the flexible and stretchable photonic crystal device. Its refractive index sensitivity is verified by simulation results, and a sensitivity of 93 nm/RIU is obtained. A flexible and stretchable photonic-crystal-based sensor shows promising in the area of tactile sensing and biosensing.

## Declarations

### Author contribution statement

Wang Peng: Conceived and designed the experiments; Performed the experiments; Analyzed and interpreted the data; Contributed reagents, materials, analysis tools or data; Wrote the paper. Bing Huang: Contributed reagents, materials, analysis tools or data; Wrote the paper. Xuanxuan Huang: Performed the experiments; Wrote the paper. Han Song: Analyzed and interpreted the data. Qingxi Liao: Contributed reagents, materials, analysis tools or data.

### Funding statement

Dr. Wang Peng was supported by Research Startup Project for New Teachers [2662020GXQD001], 10.13039/501100001809National Natural Science Foundation of China [51805194], 10.13039/501100010031Postdoctoral Research Foundation of China [2017M622411], Hubei Province Postdoctoral Foundation [2017G13], HZAU-AGIS Cooperation Fund [SZYJY2021002].

### Data availability statement

Data included in article/supp. material/referenced in article.

### Declaration of interests statement

The authors declare no conflict of interest.

### Additional information

No additional information is available for this paper.

## References

[1] Yablonovitch Eli (1987). Inhibited spontaneous emission in solid-state physics and electronics. Phys. Rev. Lett..

[2] Peng Wang (2017). CMOS-compatible fabrication for photonic crystal-based nanofluidic structure. Nanoscale Res. Lett..

[3] Akahane Yoshihiro (2003). High-Q photonic nanocavity in a two-dimensional photonic crystal. Nature.

[4] Asano Takashi (2017). Photonic crystal nanocavity with a Q factor exceeding eleven million. Opt. Express.

[5] Shen Peiyan (2021). Three-dimensional/two-dimensional photonic crystal hydrogels for biosensing. J. Mater. Chem. C.

[6] Pitruzzello Giampaolo, Krauss Thomas F. (2018). Photonic crystal resonances for sensing and imaging. J. Opt..

[7] Han Changhyun, Kang Minsu, Jeon Heonsu (2020). Lasing at multidimensional topological states in a two-dimensional photonic crystal structure. ACS Photonics.

[8] Elsayed Hussein A. (2021). Simple and efficient design towards a significant improvement of the optical absorption of amorphous silicon solar cell. J. Quant. Spectrosc. Radiat. Transf..

[9] Qutb Sameeha R., Aly Arafa H., Sabra Walied (2021). Salinity and temperature detection for seawater based on a 1D-defective photonic crystal material. Int. J. Mod. Phys. B.

[10] Taha T.A. (2022). Textured concave anti-reflecting coating and convex back reflector to enhance the absorbance of amorphous Si solar cells. Phys. Scr..

[11] Abadla Mazen M., Elsayed Hussein A., Mehaney Ahmed (2021). Novel design for the temperature sensing using annular photonic crystals. Silicon.

[12] Eid Mahmoud (2021). Mono-rectangular core photonic crystal fiber (MRC-PCF) for skin and blood cancer detection. Plasmonics.

[13] Butt M.A., Khonina S.N., Kazanskiy N.L. (2021). Recent advances in photonic crystal optical devices: a review. Opt. Laser Technol..

[14] Ahmed Ashour M., Mehaney Ahmed, Elsayed Hussein A. (2021). Detection of toluene traces in exhaled breath by using a 1D PC as a biomarker for lung cancer diagnosis. Eur. Phys. J. Plus.

[15] Mehaney Ahmed (2021). Theoretical investigations of Tamm plasmon resonance for monitoring of isoprene traces in the exhaled breath: towards chronic liver fibrosis disease biomarkers. Phys. Lett. A.

[16] Elsayed Hussein A., Sayed Fatma A., Aly Arafa H. (2021). Graphene deposited liquid crystal and thermal sensitivity using photonic crystals. Phys. Scr..

[17] Kang Christopher (2010). Photonic crystal slab sensor with enhanced surface area. Opt. Express.

[18] Cai Zhongyu (2015). Two-dimensional photonic crystal chemical and biomolecular sensors. Anal. Chem..

[19] Zhuo Yue (2014). Single nanoparticle detection using photonic crystal enhanced microscopy. Analyst.

[20] Hou Jue, Li Mingzhu, Song Yanlin (2018). Recent advances in colloidal photonic crystal sensors: materials, structures and analysis methods. Nano Today.

[21] Zhang Ya-nan (2018). Applications and developments of on-chip biochemical sensors based on optofluidic photonic crystal cavities. Lab Chip.

[22] Snapp Peter (2019). Colloidal photonic crystal strain sensor integrated with deformable graphene phototransducer. Adv. Funct. Mater..

[23] Peng Wang (2016). A nanofluidic biosensor based on nanoreplica molding photonic crystal. Nanoscale Res. Lett..

[24] Chen Jiayao (2019). Highly stretchable photonic crystal hydrogels for a sensitive mechanochromic sensor and direct ink writing. Chem. Mater..

[25] Hu Fan (2019). Gel-based artificial photonic skin to sense a gentle touch by reflection. ACS Appl. Mater. Interfaces.

[26] Liu Ya-Feng (2020). Bioinspired color-changeable organogel tactile sensor with excellent overall performance. ACS Appl. Mater. Interfaces.

[27] Zhao Pengfei (2019). Stretchable photonic crystals with periodic cylinder shaped air holes for improving mechanochromic performance. Smart Mater. Struct..

[28] Yamazaki Hiroshi, Nishiyama Michiko, Watanabe Kazuhiro (2016). Photonic Instrumentation Engineering III.

[29] Zou Liang (2017). Novel tactile sensor technology and smart tactile sensing systems: a review. Sensors.

[30] Kim Jin Tae (2018). Graphene-based optical waveguide tactile sensor for dynamic response. Sci. Rep..

[31] Chossat Jean-Baptiste, Shull Peter B. (2020). Soft acoustic waveguides for strain, deformation, localization, and twist measurements. IEEE Sens. J..

[32] Zhao Huichan (2016). Optoelectronically innervated soft prosthetic hand via stretchable optical waveguides. Sci. Robot..

[33] Peng Chang-Yi (2018). Flexible photonic crystal material for multiple anticounterfeiting applications. ACS Appl. Mater. Interfaces.

[34] Zhang Haiwen (2020). Biologically inspired flexible photonic films for efficient passive radiative cooling. Proc. Natl. Acad. Sci..

[35] Guidetti Giulia (2016). Flexible photonic cellulose nanocrystal films. Adv. Mater..

[36] Li Lan (2017). A new twist on glass: a brittle material enabling flexible integrated photonics. Int. J. Appl. Glass Sci..

[37] Gao Zhen (2018). Flexible photonic topological insulator. Adv. Opt. Mater..

[38] Cong Longqing (2017). Perovskite as a platform for active flexible metaphotonic devices. ACS Photonics.

[39] Qiao Wen (2016). Toward scalable flexible nanomanufacturing for photonic structures and devices. Adv. Mater..

[40] Kohoutek Tomas (2018). Large-area flexible colloidal photonic crystal film stickers for light trapping applications. Opt. Mater. Express.

[41] Haque Sirazul (2019). Photonic-structured TiO2 for high-efficiency, flexible and stable Perovskite solar cells. Nano Energy.

[42] Peng Wang, Liao Qingxi, Song Han (2021). A nanograting-based flexible and stretchable waveguide for tactile sensing. Nanoscale Res. Lett..

[43] Liu Jui-Nung (2011). Optimally designed narrowband guided-mode resonance reflectance filters for mid-infrared spectroscopy. Opt. Express.

[44] Block Ian D. (2008). A sensitivity model for predicting photonic crystal biosensor performance. IEEE Sens. J..

[45] Liu Jui-Nung (2014). Sculpting narrowband Fano resonances inherent in the large-area mid-infrared photonic crystal microresonators for spectroscopic imaging. Opt. Express.

[46] Peng Wang, Wu Hao (2019). Flexible and stretchable photonic sensors based on modulation of light transmission. Adv. Opt. Mater..

[47] Khanafer Khalil (2009). Effects of strain rate, mixing ratio, and stress–strain definition on the mechanical behavior of the polydimethylsiloxane (PDMS) material as related to its biological applications. Biomed. Microdevices.

[48] Peng Wang, Chen Youping, Ai Wu (2017). Higher-order mode photonic crystal based nanofluidic sensor. Opt. Commun..

[49] Bahaddur Indira (2019).

